# Screening for the Detection of *Toxoplasma gondii* IgG, IgM and IgA in Females of Reproductive Age from Western Romania

**DOI:** 10.3390/life12111771

**Published:** 2022-11-02

**Authors:** Alin Gabriel Mihu, Maria Alina Lupu, Alexandru Nesiu, Daniela Teodora Marti, Tudor Rares Olariu

**Affiliations:** 1 Discipline of Parasitology, Department of Infectious Disease, Victor Babes University of Medicine and Pharmacy, 300041 Timisoara, Romania; 2 Center for Diagnosis and Study of Parasitic Diseases, Victor Babes University of Medicine and Pharmacy, 300041 Timisoara, Romania; 3 Department of Biology and Life Sciences, Vasile Goldis University of Medicine and Pharmacy, 310025 Arad, Romania; 4 Clinical Laboratory, Institute of Cardiovascular Diseases, 300041 Timisoara, Romania; 5 Clinical Laboratory, Municipal Clinical Emergency Teaching Hospital, 300041 Timisoara, Romania

**Keywords:** *Toxoplasma gondii*, serology, screening, women of reproductive age, antibodies

## Abstract

*Toxoplasma gondii*, a zoonotic protozoan parasite, has the capacity to infect the fetus if the pregnant woman primarily acquires the infection during pregnancy. We evaluated the prevalence of *T. gondii* IgG, IgM and IgA antibodies in women of reproductive age residing in Western Romania. We also assessed the value of adding a *T. gondii* IgA test to the serologic panel for the diagnosis of toxoplasmosis, including the detection of a recently acquired infection. Serologic testing to demonstrate the presence of *T. gondii* IgG antibodies was conducted in 1317 females aged 15–45 years. *T. gondii* IgM and IgA antibody tests were performed in those with detectable IgG antibodies and IgG avidity test was performed if IgM and/or IgA screening test results were positive. *T. gondii* IgG were detected in 607 (46.09%; 95%CI: 43.41–48.79) of 1317 study participants and IgG seroprevalence tended to increase with age from 35.44% (95%CI: 29.89–41.30) in age group 15–24 years to 62.85% (95%CI: 56.57–68.82) in age group 35–45 years, showing a significant age-associated increase (*p* < 0.001). Of the 607 persons with detectable *T. gondii* IgG antibodies, *T. gondii* IgM antibodies were demonstrated in 8.90% (95%CI: 6.88–11.43), *T. gondii* IgA in 1.65% (95%CI: 0.90–3.01) and both *T. gondii* IgM and IgA in 0.99% (95%CI: 0.45–2.14). The prevalence of IgA antibodies tended to decrease with increasing avidity, from 75% (95%CI: 19.41–99.37) in samples with low avidity to 11.76% (95%CI: 4.44–23.87) in those with high avidity (*p* = 0.01). Of the study participants who were positive for both *T. gondii* IgM and IgA antibodies, 66.67% had low or equivocal IgG avidity test results compared to 6.25% who tested positive for IgM, were negative for IgA and in whom low or equivocal IgG avidity test results were noted (*p* = 0.001). This study indicates that in Western Romania, *T. gondii* IgG seroprevalence is high in females of reproductive age and *T. gondii* IgA antibodies may be rarely detected during a serologic screening. However, in individuals with demonstrable *T. gondii* IgG and IgM antibodies, testing for *T. gondii* IgA may improve the rate for the detection of a recently acquired toxoplasmosis.

## 1. Introduction

*Toxoplasma gondii*, a zoonotic protozoan parasite with a worldwide distribution, can infect both humans and all warm-blooded animals, including mammals and birds [1,2]. Felids (domestic and wild cats), the only definitive hosts, harbor the sexual parasitic cycle and spread oocysts through feces [2,3]. Asexual replication of *T. gondii* occurs in a wide variety of vertebrates which serve as intermediate hosts [4].

*T. gondii*, by crossing the placenta, has the capacity to infect the fetus if the pregnant woman primarily acquires the infection during pregnancy [5]. In congenitally infected infants, the severity of fetal damage is related inversely to the stage of pregnancy when maternal infection occurs: manifestations are usually more severe if *T. gondii* is transmitted early in the gestation period [2,6] and spontaneous abortion, prematurity or stillbirth may result [7]. Chorioretinitis, intracranial calcifications, hydrocephalus, microcephalus, convulsions, hepatosplenomegaly, jaundice or fever are among the most frequently clinical signs reported in infants with congenital toxoplasmosis [7,8,9]. The global prevalence of latent infection with *T. gondii* in pregnant women is estimated at 33.8% and varies widely from 0.7% in South Korea to 92% in Ghana. In European countries, the prevalence rate is 31.2% [10]. Approximately 1.1% of pregnant women are acutely infected with *T. gondii* during pregnancy worldwide, with a prevalence of 0.5% reported in European regions, suggesting that a considerable number of fetuses are at risk of acquiring the infection [11]. Given the severe clinical manifestations that *T. gondii* may cause in these patients, it is important to survey its distribution in females of reproductive age and pregnant women.

In Europe, data regarding congenitally infected children are still limited, given that a nationwide epidemiological surveillance system for congenital toxoplasmosis has been implemented only in France and Germany, and in Italy at a regional level [12]. The rate of congenital toxoplasmosis in Romania is unknown [13] and little information is available regarding *T. gondii* seroprevalence in children [14], pregnant women [5] or women of reproductive age [15].

Diagnosis of both acquired and congenital toxoplasmosis is still based on serological methods [6,16]. The evaluation of *T. gondii* antibodies (IgG, IgM, IgA) and IgG avidity test results usually allows physicians to assess the immunologic status of a patient and to diagnose seroconversion [17,18,19], but the serological diagnosis of toxoplasmosis is complex and the interpretation of test results may be complicated by the long-term persistence of specific IgM [17]. The IgG avidity test can discriminate between past and recently acquired infections: a low avidity IgG test result is in general suggestive of a recent infection and a high avidity test result excludes a recent infection in the past 4 months [6,16]. Some laboratories use IgA test as an additional marker for diagnosis of acute toxoplasmosis [20] and recent surveys have demonstrated that *T. gondii* IgA antibody testing might represent a valuable adjunct for determining the timing of the infection, as part of a reference panel for the diagnosis of acute toxoplasmosis [21]. Moreover, *T. gondii* IgA was sometimes useful in detecting a recently acquired infection in the absence of *T. gondii* IgM antibodies [21,22].

Therefore, in this study, we assessed the prevalence of *T. gondii* IgG, IgM and IgA antibodies in women of childbearing age residing in Western Romania. We also evaluated the value of adding *T. gondii* IgA test to the serologic screening for toxoplasmosis, including the detection of a recently acquired infection.

## 2. Materials and Methods

### 2.1. Study Design

Venous blood samples were collected between 1 February 2018 and 1 February 2019, from 1317 consecutive women of reproductive age (15–45 years), presented for routine check-up, at the Municipal Clinical Emergency Hospital Outpatient Clinic in Timisoara, Romania. Females were residents of Timis county, located in Western Romania, with a total population of 705,113. The age given for each study participant is the age when the blood sample was drawn upon enrolment in the study. Study participants were grouped according to their age in 3 age groups: 15–24 years, 25–34 years and 35–45 years. Peripheral blood samples were collected by venipuncture in 6 mL red-top (plain, non-serum separator) tubes and the serum samples were tested for the presence of specific IgG anti-*T. gondii* antibodies. In cases of positive results, serum samples were further transferred to 1.5 mL Eppendorf tubes and stored at −20 °C until *T. gondii* IgM and IgA antibody tests were performed. A specific IgG avidity test was performed only when the presence of *T. gondii* IgM and/or IgA antibodies was confirmed.

No clinical criteria were used to include subjects in this study.

### 2.2. Serologic Tests

All serum samples were assessed for anti-*T. gondii* IgG with the ADVIA Centaur^®^ XP (Siemens Healthcare Diagnostics, Erlangen, Germany) electrochemiluminescence *Toxo* IgG assay. The sensitivity and specificity for IgG detection were both 100% [23].

Enzyme-linked fluorescent assay (ELFA) designed for VIDAS (bioMérieux, Marcy-l’Etoile, France) was used for identification of serum anti-*T. gondii* IgM antibodies and evaluation of IgG avidity (VIDAS *Toxo* IgM kit and *Toxo* IgG Avidity kit, respectively). ELFA IgM has a sensitivity of 100% and a specificity of 98.6% [24,25]. IgG avidity Vidas was recently found to have an accuracy of 93.4% in detecting a *T. gondii* infection dating more than 4 months [26].

Serum anti-*T. gondii* IgA antibodies were determined using a solid phase enzyme-linked immunosorbent assay (ELISA) (DRG *Toxoplasma gondii* IgA ELISA kit, DRG, Marburg, Germany) with a diagnostic sensitivity and specificity of 100% [27].

All tests were carried out according to manufacturers’ recommendations regarding calibration and the running of controls.

### 2.3. Interpretation of the Serologic Test Results

Interpretation of test results was based on each of the manufacturer’s criteria.

*T. gondii* IgG antibody test results were interpreted as follows: <6.40 IU/mL, negative; ≥6.4 to 10 IU/mL, equivocal; >10 IU/mL, positive [28]. *T. gondii* IgM test results were interpreted as follows: <0.55, negative; ≥0.55 to 0.65, equivocal; >0.65, positive [29]. *T. gondii* IgA test results were interpreted as negative if the ratio was <0.9; equivocal between 0.9 and 1.0 and positive if the ratio was >1.1 [27]. For the purposes of this study, IgG, IgM and IgA equivocal test results were considered negative.

The Vidas IgG avidity test was interpreted as follows: <0.2, low avidity; ≥0.2 to 0.29, equivocal result; ≥0.3%, high avidity [29]. Low or equivocal tests results indicate the possibility that an infection occurred within the past 4 months, and a high test result excludes the possibility of a primary infection within the previous 4 months [21].

### 2.4. Data Management and Statistical Analysis

Data were collected using a Microsoft Excel database, version 2011 (Microsoft Corp., Redmond, WA, USA), and the statistical analyses were performed using Epi Info statistical package, version 3.3.2 (Centers for Disease Control and Prevention, Atlanta, GA, USA) and MedCalc for Windows, version 19.4 (MedCalc Software, Ostend, Belgium). Data are presented as number (percentage), mean ± standard deviation (SD), odds ratio (OR) with 95% confidence interval (CI). Mantel–Haenszel chi-square test and Fisher’s 2-tailed exact test were used to compare proportions between groups. A *p* value of <0.05 was considered of statistical significance.

### 2.5. Ethical Consideration

This study was approved by the Victor Babes University Ethics Committee, Timisoara, Romania (no.2 from 8 January 2018). All participants included in the study were thoroughly informed about the study goals and the procedures, and provided written informed consent. For individuals under the age of eighteen, the parents/legal guardians provided the written informed consent.

## 3. Results

Of the 1317 female study participants aged 15–45 years (mean = 29.45 ± 6.16 years), *T. gondii* IgG antibodies were demonstrated in 607 (46.09%) (95%CI: 43.41–48.78) and tended to increase with age, from 35.44% (95%CI: 29.89–41.30) in age group 15–24 years to 62.85% (95%CI: 56.57–68.82) in age group 35–45 years, showing a significant age-associated increase (*p* < 0.001; OR = 1.73; 95%CI:1.46–2.06) (Table 1).

**Figure 1:**
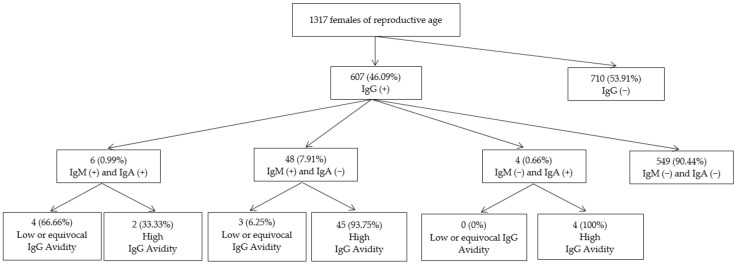
Serologic screening for the detection of *Toxoplasma gondi i* IgG, IgM and IgA antibodies in 1317 females of reproductive age from Western Romania.

*T. gondii* IgM and IgA antibody tests were performed for the 607 females identified with IgG antibodies and the IgG avidity test was performed subsequently in 58 (9.56%) samples that tested positive for *T. gondii* IgM and/or IgA antibodies. In 549 (90.44%) of the 607 cases in which the IgG avidity test was not performed, the diagnosis of chronic infection was based on the negative results for both *T. gondii* IgM and IgA antibodies (Figure 1).

*T. gondii* IgM antibodies were demonstrated in 54 (8.90%; 95%CI: 6.88–11.43)) of the 607 IgG positive study participants, IgA in 10 (1.65%; 95%CI: 0.90–3.01) and both *T. gondii* IgM and IgA in 6 (0.99%; 95%CI: 0.45–2.14). Of the 54 females with IgM antibodies, only 6 (11.11%) also had IgA antibodies, whereas 6/10 (60%) of study participants with IgA antibodies also had IgM antibodies (Table 2, Figure 1).

Of the 58 serum samples that tested for IgG avidity, 7 (12.07%) had low or equivocal IgG avidity test results: 4 (6.90%) had low IgG avidity test results and 3 (5.17%) had equivocal IgG avidity test results (Figure 1). Of the 6 study participants who were positive for both *T. gondii* IgM and IgA antibodies, 4 (66.67%) had low or equivocal IgG avidity test results compared to 3 of 48 (6.25%) who tested positive for IgM, were negative for IgA and in whom low or equivocal IgG avidity test results were noted (*p* = 0.001) (Table 3, Figure 1).

The prevalence of IgA antibodies tended to decrease with increasing avidity, from 75% (3/4) in samples with low avidity (0.0–0.19) to 11.76% (6/51) with high avidity (≥0.3) (*p* = 0.01).

Moreover, of the 51 females with a high avidity test result (≥0.3), only 11.76% (6/51) had IgA antibodies compared to 92.16% (47/51) of those in whom IgM antibodies were detected (*p* < 0.001) (Table 4).

## 4. Discussion

In immunocompetent individuals, infection with *T. gondii* is generally self-limiting and asymptomatic [30,31], but is capable of causing devastating congenital infection by vertical transmission [32]. The maternal–fetal transmission rate varies from 2.6% at 3 weeks to 69.8% at 39 weeks of pregnancy [33]. Serologic screening of pregnant women followed by treatment in specific cases can reduce the rates of vertical transmission and therefore the disease severity in the affected fetus [32,33,34,35,36].

There is little information available to the international scientific community regarding *T. gondii* prevalence in Romanian women. The studies published so far have been carried out on small samples of population and revealed a seroprevalence from 19.5% in girls aged 1–18 years [14], to 55.8% in pregnant women aged 12–41 years [5], based on serologic test results for IgG and/or IgM anti-*T. gondii* antibodies. The 46.09% seroprevalence of *T. gondii* IgG in our study group is lower than the 57.6% reported by Olariu et al. in 2008 [15] in the same geographical area and similar to the 43.2% *T. gondii* prevalence recently demonstrated in female blood donors aged between 18 and 45 years from Western Romania [37]. These results suggest a decreasing trend in the seroprevalence of *T. gondii* in this region, and are consistent with results of previous studies conducted in Europe that confirmed such a decrease in seroprevalence [38,39,40]. There are several factors which can explain this decline: nowadays domestic cats feed less often with raw meat (rodents hunting) because they are fed with dry food [41]; crude vegetables are washed before consumption [42]; the quality of water has improved and the consumption of bottled water has increased [42]; commercialized meat is infrequently infected with *T. gondii*; during transportation, meat is deep-freezed and this can lead to a decrease of consumption of infected meat [42].

In this study, the prevalence of IgA antibodies was 1.65% compared to the 8.90% prevalence of IgM, suggesting that the detection rate of *T. gondii* IgA antibodies may be lower than the IgM, during a serologic screening for toxoplasmosis. The prevalence of *T. gondii* IgA antibodies found in our survey is lower than the 13% and 8.7% seroprevalence reported by investigators in pregnant women from the USA [21] and in mothers with spontaneous abortion from Iran [43], respectively. Unlike our study, where females were investigated in the order in which they presented for routine laboratory check-up, in other studies participants were pregnant women suspected to have been infected with *T. gondii*. However, in Brazilian pregnant women selected by convenience when they presented for the antenatal care consultation, the seroprevalence of *T. gondii* IgA antibodies was 0.82% [44], lower than our findings. These differences may also be explained by different sample size and various assays (with different sensitivities and/or specificities) used to identify the presence of *T. gondii* IgA antibodies [37].

When conventional serologic tests (*T. gondii* IgG, IgM and IgA) were used in our study group, 9.56% (58/607) of females could be suspected to have been recently infected. If IgG avidity and serologic tests (IgG, IgM, IgA) were combined, only 1.15% (7/607) of the females could be suspected of having a recent infection. Similar results were published by Berredjem et al. [45].

Results of this survey suggest that a recent infection is more likely to be diagnosed in women with both *T. gondii* IgM and IgA antibodies (66.67%) compared to women in whom only IgM antibodies were present (6.25%). This indicates that *T. gondii* IgA is more likely to be positive if the infection occurred more recently, similar with results of previous studies [21,43,45,46]. To assess the risk of active disease, especially in pregnant women, testing for *T. gondii* IgG and IgM antibodies alone is often insufficient [47,48], because *T. gondii* IgM, when detected, may also indicate a long-term persistence of these specific antibodies [49]. However, the presence of *T. gondii* IgA antibodies at the same time with IgM and IgG is in favor of acute infection, given that the IgA test has a higher specificity and positive predictive value for the diagnosis of acute infection [20].

Though our sample size was large, the number of individuals tested for avidity was small and this may be considered a study limitation. However, our assessments regarding the interpretation of *T. gondii* IgM, IgA and IgG avidity test results are in line with those recently reported in a study performed in the USA [21]. The higher prevalences of IgM and IgA antibodies found in the USA reference laboratory may be explained by the referral bias, due to testing of pregnant women on the doctors’ recommendation in case of suspected *T. gondii* infection, compared to the lower IgM and IgA values found in our study where testing was performed by screening among women of reproductive age. In addition, serum samples with negative results for *T. gondii* IgG antibodies were not tested further for the presence of specific *T. gondii* IgM antibodies, and this may also be listed as a limitation of our study. However, cases with detectable IgM antibodies in absence of IgG antibodies are extremely rare [50] and it is highly unlikely that IgM testing of negative IgG sera may significantly change the results of this study.

In most countries, efforts at preventing *T. gondii* infection frequently focus on pregnant women in order to reduce the risk of miscarriage and congenital infections. However, outbreaks in immunocompetent individuals have been described, which suggests that prevention and control measures, together with health education should also be directed to the general population [51].

## 5. Conclusions

Results of the present study suggest that *T. gondii* prevalence is still high in Western Romania, although a declining trend of the seroprevalence was noted.

This survey indicates that *T. gondii* IgA antibodies may be rarely detected in females living in a Romanian endemic region and in whom a serologic screening has been performed. Therefore, our data suggest that *T. gondii* IgA test should not be routinely performed for screening purposes. However, in persons with demonstrable *T. gondii* IgG and IgM antibodies, testing for *T. gondii* IgA may improve the rate for the detection of a recently acquired toxoplasmosis.

## Figures and Tables

**Table 1:** Seroprevalence of *Toxoplasma gondii* IgG in females of reproductive age from Western Romania according to age.

		Prevalence of *T. gondii* Infection Univariate Analysis		
Variables	No. Tested	N (%)	OR (95% CI)	*p* Value
Age groups (years)				
15–24	285	101 (35.44)	1 (Ref.)	
25–34	779	347 (44.54)	1.46 (1.11–1.94)	0.007
35–45	253	159 (62.85)	3.08 (2.17–4.38)	<0.001
Total	1317	607 (46.09)		-

N, number of *T. gondii* seropositive females; OR, odds ratio; CI, confidence interval; Ref., reference.

**Table 2:** *T. gondii* IgM and IgA antibody test results in females with detectable *T. gondii* IgG antibodies.

Result(s) for Individual Serum Samples	No. (%) of Females Aged 15–45 Years with Detectable *T. gondii* IgG Antibodies
IgM (+)	54 (8.90)
IgM (−)	553 (91.10)
IgA (+)	10 (1.65)
IgA (−)	597 (98.35)
IgM (+), IgA (+)	6 (0.99)
IgM (+), IgA (−)	48 (7.91)
IgM (−), IgA (+)	4 (0.66)
IgM (−), IgA (−)	549 (90.44)

(+), positive; (−), negative.

**Table 3:** *Toxoplasma gondii* IgM and IgA antibody test results compared to IgG avidity results in females with detectable *Toxoplasma gondii* IgG antibodies.

Serologic Tests	IgG Avidity Assay		No. of Samples Tested
	Low or Equivocal	High	
	No. (%) of Samples	No. (%) of Samples	
IgM (+)	7 (12.96)	47 (87.04)	54
IgM (−)	0 (0)	4 (100)	4
IgA (+)	4 (40)	6 (60)	10
IgA (−)	3 (6.25)	45 (93.75)	48
IgM (+), IgA (+)	4 (66.67)	2 (33.33)	6
IgM (+), IgA (−)	3 (6.25)	45 (93.75)	48
IgM (−), IgA (+)	0 (0)	4 (100)	4

(+), positive; (−), negative.

**Table 4:** *Toxoplasma gondii* IgM and IgA antibody test results compared to IgG avidity results.

IgG Avidity	IgG Avidity Test Interpretation	No. of Females Tested	No. (%) of Females Tested Positive	
			IgM	IgA
0.0–0.19	low avidity	4	4 (100)	3 (75)
0.20–0.29	equivocal	3	3 (100)	1 (33.33)
≥0.3	high avidity	51	47 (92.16)	6 (11.76)

## Data Availability

Datasets used and/or analyzed during the current study are available from the corresponding author on reasonable request.

## References

[1] Dubey J.P. (2008). The History of Toxoplasma Gondii—The First 100 Years. J. Eukaryot. Microbiol..

[2] Robert-Gangneux F., Dardé M.-L. (2012). Epidemiology of and Diagnostic Strategies for Toxoplasmosis. Clin. Microbiol. Rev..

[3] Mendez O.A., Koshy A.A. (2017). Toxoplasma Gondii: Entry, Association, and Physiological Influence on the Central Nervous System. PLoS Pathog..

[4] Sibley L.D., Khan A., Ajioka J.W., Rosenthal B.M. (2009). Genetic Diversity of Toxoplasma Gondii in Animals and Humans. Philos. Trans. R. Soc. Lond. B. Biol. Sci..

[5] Olariu T.R., Ursoniu S., Hotea I., Dumitrascu V., Anastasiu D., Lupu M.A. (2020). Seroprevalence and Risk Factors of Toxoplasma Gondii Infection in Pregnant Women from Western Romania. Vector Borne Zoonotic Dis. Larchmt. N.

[6] Furtado J.M., Smith J.R., Belfort R., Gattey D., Winthrop K.L. (2011). Toxoplasmosis: A Global Threat. J. Glob. Infect. Dis..

[7] McAuley J.B. (2014). Congenital Toxoplasmosis. J. Pediatr. Infect. Dis. Soc..

[8] Olariu T.R., Remington J.S., McLeod R., Alam A., Montoya J.G. (2011). Severe Congenital Toxoplasmosis in the United States: Clinical and Serologic Findings in Untreated Infants. Pediatr. Infect. Dis. J..

[9] Olariu T.R., Press C., Talucod J., Olson K., Montoya J.G. (2019). Congenital Toxoplasmosis in the United States: Clinical and Serologic Findings in Infants Born to Mothers Treated during Pregnancy. Parasite Paris Fr..

[10] Rostami A., Riahi S.M., Gamble H.R., Fakhri Y., Nourollahpour Shiadeh M., Danesh M., Behniafar H., Paktinat S., Foroutan M., Mokdad A.H. (2020). Global prevalence of latent toxoplasmosis in pregnant women: A systematic review and meta-analysis. Clin. Microbiol. Infect..

[11] Rostami A., Riahi S.M., Contopoulos-Ioannidis D.G., Gamble H.R., Fakhri Y., Shiadeh M.N., Foroutan M., Behniafar H., Taghipour A., Maldonado Y.A. (2019). Acute Toxoplasma infection in pregnant women worldwide: A systematic review and meta-analysis. PLoS Negl. Trop. Dis..

[12] Fanigliulo D., Marchi S., Montomoli E., Trombetta C.M. (2020). Toxoplasma Gondii in Women of Childbearing Age and during Pregnancy: Seroprevalence Study in Central and Southern Italy from 2013 to 2017. Parasite Paris Fr..

[13] Dubey J.P., Hotea I., Olariu T.R., Jones J.L., Dărăbuş G. (2014). Epidemiological Review of Toxoplasmosis in Humans and Animals in Romania. Parasitology.

[14] Căpraru I.D., Lupu M.A., Horhat F., Olariu T.R. (2019). Toxoplasmosis Seroprevalence in Romanian Children. Vector Borne Zoonotic Dis. Larchmt. N.

[15] Olariu T.R., Darabus G., Cretu O., Jurovits O., Erdelean V., Marincu I., Iacobiciu I., Petrescu C., Koreck A. (2008). Prevalence of Toxoplasma Gondii Antibodies among Women of Childbearing Age in Timis Country. Lucr. Stiintifice Med. Vet. Timisoara.

[16] Halonen S.K., Weiss L.M. (2013). Toxoplasmosis. Handb. Clin. Neurol..

[17] Fricker-Hidalgo H., Cimon B., Chemla C., Darde M.L., Delhaes L., L’ollivier C., Godineau N., Houze S., Paris L., Quinio D. (2013). Toxoplasma Seroconversion with Negative or Transient Immunoglobulin M in Pregnant Women: Myth or Reality? A French Multicenter Retrospective Study. J. Clin. Microbiol..

[18] Jones J.L., Lopez A., Wilson M., Schulkin J., Gibbs R. (2001). Congenital Toxoplasmosis: A Review. Obstet. Gynecol. Surv..

[19] Khurana S., Batra N. (2016). Toxoplasmosis in Organ Transplant Recipients: Evaluation, Implication, and Prevention. Trop. Parasitol..

[20] Li X., Pomares C., Gonfrier G., Koh B., Zhu S., Gong M., Montoya J.G., Dai H. (2016). Multiplexed Anti-Toxoplasma IgG, IgM, and IgA Assay on Plasmonic Gold Chips: Towards Making Mass Screening Possible with Dye Test Precision. J. Clin. Microbiol..

[21] Olariu T.R., Blackburn B.G., Press C., Talucod J., Remington J.S., Montoya J.G. (2019). Role of Toxoplasma IgA as Part of a Reference Panel for the Diagnosis of Acute Toxoplasmosis during Pregnancy. J. Clin. Microbiol..

[22] Liu Q., Wang Z.-D., Huang S.-Y., Zhu X.-Q. (2015). Diagnosis of Toxoplasmosis and Typing of Toxoplasma Gondii. Parasit. Vectors.

[23] Villard O., Cimon B., L’Ollivier C., Fricker-Hidalgo H., Godineau N., Houze S., Paris L., Pelloux H., Villena I., Candolfi E. (2016). Help in the Choice of Automated or Semiautomated Immunoassays for Serological Diagnosis of Toxoplasmosis: Evaluation of Nine Immunoassays by the French National Reference Center for Toxoplasmosis. J. Clin. Microbiol..

[24] Wilson M., Remington J.S., Clavet C., Varney G., Press C., Ware D. (1997). Evaluation of Six Commercial Kits for Detection of Human Immunoglobulin M Antibodies to Toxoplasma Gondii. The FDA Toxoplasmosis Ad Hoc Working Group. J. Clin. Microbiol..

[25] Gharavi M.J., Oormazdi H., Roointan E.S. (2008). A Comparative Study on Sensitivity and Specificity of Conventional and Unconventional IgG and IgM Assays for Diagnosis of Toxoplasmosis. Iran. J. Public Health.

[26] Smets A., Fauchier T., Michel G., Marty P., Pomares C. (2016). Comparison of *Toxoplasma gondii* IgG Avidity Architect and Vidas Assays with the Estimated Date of Infection in Pregnant Women. Parasite Paris Fr..

[27] Toxoplasma Gondii IgA ELISA EIA-3683, Version 3.0. https://www.drg-diagnostics.de/files/eia-3683_ifu--toxoplasma-gondii-iga_2017-10-10_ce_endeites.pdf.

[28] Robert-Gangneux F., Guegan H. (2021). Anti-Toxoplasma IgG assays: What performances for what purpose? A systematic review. Parasite.

[29] Murat J.B., Dard C., Fricker Hidalgo H., Dardé M.L., Brenier-Pinchart M.P., Pelloux H. (2013). Comparison of the Vidas system and two recent fully automated assays for diagnosis and follow-up of toxoplasmosis in pregnant women and newborns. Clin. Vaccine Immunol..

[30] Montoya J.G., Liesenfeld O. (2004). Toxoplasmosis. Lancet Lond. Engl..

[31] Cañedo-Solares I., Gómez-Chávez F., Luna-Pastén H., Ortiz-Alegría L.B., Flores-García Y., Figueroa-Damián R., Macedo-Romero C.A., Correa D. (2018). What Do Anti-Toxoplasma Gondii IgA and IgG Subclasses in Human Saliva Indicate?. Parasite Immunol..

[32] El Bissati K., Levigne P., Lykins J., Adlaoui E.B., Barkat A., Berraho A., Laboudi M., El Mansouri B., Ibrahimi A., Rhajaoui M. (2018). Global Initiative for Congenital Toxoplasmosis: An Observational and International Comparative Clinical Analysis. Emerg. Microbes Infect..

[33] Wallon M., Peyron F., Cornu C., Vinault S., Abrahamowicz M., Kopp C.B., Binquet C. (2013). Congenital Toxoplasma Infection: Monthly Prenatal Screening Decreases Transmission Rate and Improves Clinical Outcome at Age 3 Years. Clin. Infect. Dis. Off. Publ. Infect. Dis. Soc. Am..

[34] Chaudhry S.A., Gad N., Koren G. (2014). Toxoplasmosis and Pregnancy. Can. Fam. Physician Med. Fam. Can..

[35] Guegan H., Stajner T., Bobic B., Press C., Olariu R.T., Olson K., Srbljanovic J., Montoya J.G., Djurković-Djaković O., Robert-Gangneux F. (2021). Maternal Anti-Toxoplasma Treatment during Pregnancy Is Associated with Reduced Sensitivity of Diagnostic Tests for Congenital Infection in the Neonate. J. Clin. Microbiol..

[36] Augustine S.A.J. (2016). Towards Universal Screening for Toxoplasmosis: Rapid, Cost-Effective, and Simultaneous Detection of Anti-Toxoplasma IgG, IgM, and IgA Antibodies by Use of Very Small Serum Volumes. J. Clin. Microbiol..

[37] Lupu M.A., Lighezan R., Paduraru A.A., Dragomir A., Pavel R., Grada S., Mihu A.G., Ursoniu S., Olariu T.R. (2022). Seroepidemiology of Toxoplasma Gondii Infection in Blood Donors from Western Romania. Microorganisms.

[38] Berger F., Goulet V., Le Strat Y., Desenclos J.-C. (2009). Toxoplasmosis among Pregnant Women in France: Risk Factors and Change of Prevalence between 1995 and 2003. Rev. Epidemiol. Sante Publique.

[39] Nogareda F., Le Strat Y., Villena I., De Valk H., Goulet V. (2014). Incidence and Prevalence of Toxoplasma Gondii Infection in Women in France, 1980–2020: Model-Based Estimation. Epidemiol. Infect..

[40] Gargaté M.J., Ferreira I., Vilares A., Martins S., Cardoso C., Silva S., Nunes B., Gomes J.P. (2016). Toxoplasma Gondii Seroprevalence in the Portuguese Population: Comparison of Three Cross-Sectional Studies Spanning Three Decades. BMJ Open.

[41] Afonso E., Germain E., Poulle M.-L., Ruette S., Devillard S., Say L., Villena I., Aubert D., Gilot-Fromont E. (2013). Environmental Determinants of Spatial and Temporal Variations in the Transmission of Toxoplasma Gondii in Its Definitive Hosts. Int. J. Parasitol. Parasites Wildl..

[42] Guigue N., Léon L., Hamane S., Gits-Muselli M., Le Strat Y., Alanio A., Bretagne S. (2018). Continuous Decline of Toxoplasma Gondii Seroprevalence in Hospital: A 1997–2014 Longitudinal Study in Paris, France. Front. Microbiol..

[43] Amin A., Mazloomzadeh S., Haniloo A., Mohammadian F., Fazaeli A. (2012). Evaluation of Anti-Toxoplasma IgG, IgM, and IgA in Mothers with Spontaneous Abortion in Zanjan, Northwest Iran. Korean J. Parasitol..

[44] Gontijo da Silva M., Clare Vinaud M., de Castro A.M. (2015). Prevalence of Toxoplasmosis in Pregnant Women and Vertical Transmission of Toxoplasma Gondii in Patients from Basic Units of Health from Gurupi, Tocantins, Brazil, from 2012 to 2014. PLoS ONE.

[45] Berredjem H., Aouras H., Benlaifa M., Becheker I., Djebar M.R. (2017). Contribution of IgG Avidity and PCR for the Early Diagnosis of Toxoplasmosis in Pregnant Women from the North-Eastern Region of Algeria. Afr. Health Sci..

[46] Murata F.H.A., Ferreira M.N., Camargo N.S., Santos G.S., Spegiorin L.C.J.F., Silveira-Carvalho A.P., Pereira-Chioccola V.L., de Mattos L.C., de Mattos C.C.B. (2016). Frequency of Anti- Toxoplasma Gondii IgA, IgM, and IgG Antibodies in High-Risk Pregnancies, in Brazil. Rev. Soc. Bras. Med. Trop..

[47] Foudrinier F., Marx-Chemla C., Aubert D., Bonhomme A., Pinon J.M. (1995). Value of Specific Immunoglobulin A Detection by Two Immunocapture Assays in the Diagnosis of Toxoplasmosis. Eur. J. Clin. Microbiol. Infect. Dis. Off. Publ. Eur. Soc. Clin. Microbiol..

[48] Stepick-Biek P., Thulliez P., Araujo F.G., Remington J.S. (1990). IgA Antibodies for Diagnosis of Acute Congenital and Acquired Toxoplasmosis. J. Infect. Dis..

[49] Liesenfeld O., Montoya J.G., Kinney S., Press C., Remington J.S. (2001). Effect of Testing for IgG Avidity in the Diagnosis of Toxoplasma Gondii Infection in Pregnant Women: Experience in a US Reference Laboratory. J. Infect. Dis..

[50] Jones J.L., Kruszon-Moran D., Elder S., Rivera H.N., Press C., Montoya J.G., McQuillan G.M. (2018). Toxoplasma gondii Infection in the United States, 2011-2014. Am. J. Trop. Med. Hyg..

[51] Pinto-Ferreira F., Caldart E.T., Pasquali A.K.S., Mitsuka-Breganó R., Freire R.L., Navarro I.T. (2019). Patterns of Transmission and Sources of Infection in Outbreaks of Human Toxoplasmosis. Emerg. Infect. Dis..

